# Effect of Combined Sorafenib/Cisplatinum Treatment on the Autophagy and Proliferation of Hepatocellular Carcinoma hepG2 Cells *in Vitro*

**DOI:** 10.31557/APJCP.2020.21.10.2853

**Published:** 2020-10

**Authors:** Yaoting Wang, Lei Wang

**Affiliations:** 1 *Department of Oncology, Dongying People’s Hospital, Dongying, China. *; 2 *Department of Oncology, Yantaishan Hospital-Yantai, China. *

**Keywords:** Sorafenib, cis-platinum, autophagy, hepatocellular carcinoma

## Abstract

**Objective:**

To explore the effect of combined Sorafenib/ cisplatinum treatment on the autophagy and proliferation of hepatocellular carcinoma (HepG2) cells in vitro.

**Methods:**

HepG2 cells were cultured and treated with different concentrations of Sorafenib, cisplatinum, or a combination of both over a 24-hour period. Cell proliferation was evaluated using a CCK8 assay, and the mRNA expression of the autophagy-related proteins AKT, mTOR, and LC3 were detected using quantitative PCR (qPCR). AKT, pAKT (Ser473), mTOR, pmTOR (Ser2448), LC3I, and LC3II protein expression levels were evaluated by western blot.

**Results:**

We found that the survival rate of HepG2 cells was 47.42% when treated with Sorafenib (10 μmol/L) monotherapy, and 46.04% when treated with cisplatinum (10 mg/L) monotherapy. When Sorafenib(10 μmol/L) was combined with cisplatinum (10 mg/L), the cellular proliferation and survival rate was only 16.71% ( P <0.05). qPCR and western blot revealed that a combination of Sorafenib (10 μmol/L) and cisplatinum (10 mg/L) reduced the transcription and protein expression of autophagy-related AKT and mTOR but increased that of LC3 (P <0.05).

**Conclusion:**

Combining Sorafenib and cisplatinum can effectively induce cell autophagy and reduce cellular proliferation via the PI3K/AKT/mTOR signal pathway.

## Introduction

Hepatocellular carcinoma (HCC is increasing in prevalence with the incidence of HCC in China making up nearly half of the global HCC cases every year. Most patients are diagnosed with terminal-stage HCC, as early stage symptoms tend to be mild and difficult to identify. The conventional clinical treatment of HCC includes surgery, chemotherapy, radiotherapy, interventional therapy and targeted therapy. Currently, HCC is preferentially treated using a surgery based, comprehensive treatment plan (Lau et al., 2014). However, these treatments also have their drawbacks (Chen and Zhu, 2016; Lau et al., 2014; Gedaly et al., 2013 ).Cell autophagy is a conservative mechanism used to maintain homeostasis which can be impaired by aging, with excessive cellular autophagy often resulting in uncontrolled cell death. The PI3K/AKT/mTOR signaling pathway is a critical component in HCC mediated autophagy, making it an ideal therapeutic target. Sorafenib is a multi-kinase inhibitor of more than a dozen kinases, including serine/threonine kinase Raf, proangiogenic receptor tyrosine kinase VEGFR, and platelet-derived growth factor receptor (PDGFR) (Chaparro et al., 2008). Moreover, Sorafenib can inhibit cell proliferation via alterations in PI3K/AKT/mTOR signaling (He et al., 2015). Sorafenib can dramatically extend the median survival duration and the time to progression for the late or advanced stage HCC and, at present, Sorafenib is the only effective drug for targeted HCC treatment. Cis-platinum has been used as a first-line treatment in many solid tumors, with this compound inhibiting cellular proliferation by interacting with the DNA of tumor cells (Zou et al., 2012). The aim of this study was to evaluate the effect of a combined Sorafenib/cisplatinum therapeutic on HCC proliferation and autophagy, and to determine their effect on PI3K/AKT/mTOR signaling.

## Materials and Methods

*Materials*

The human HCC cell line, HepG2, was a gift from the Department of Microbiology at Qingdao University, China. Primary antibodies against AKT, phospho-AKT (ser473), mTOR, phospho-mTOR (Ser2448), LC3I, LC3II, and all the secondary antibodies were obtained from Cell Signaling Technology (USA) and Proteintech Group Inc (China). Protein markers and other chemicals used in the western blots were also obtained from Cell Signaling Technology. The Cell Counting Kit (CCK-8/WST-8) was purchased from BOSTER biology technology co., LTD (USA). All of the chemicals for fluorescent quantitative Polymerase Chain Reaction (qPCR) were obtained from Thermo Fisher Scientific (USA). Sorafenib and cisplatinum were bought from Selleck.

*Methods*

*Cell culture*

The human HCC cell line, HepG2, was cultured in Hyclone RPMI1640 medium supplemented with 15% heat inactivated fetal bovine serum (FBS) in a 37^o^C incubator with 5% CO_2_ .

*Cell proliferation assays*

HepG2 cells were plated in 96-well culture dishes at 1×10^4^ cells/mL in 100 μl Hyclone RPMI1640 supplemented with 15% FBS and cultured for 24 h. The cells were then treated with Sorafenib (5, 10, 15, 20 μmol/L), Cisplatinum (5, 10, 15 and 20 mg/L), or a combination of Sorafenib (10 μmol/L) and Cisplatinum (10 mg/L) for 24 h. Untreated cells were used as the blank control. A total of 10 μl of the CCK8 reagent was then added to each well and cultured for a further 2 h at 37^o^C. Then we measured the absorbance of each well at 450 nm.

*Quantitative PCR*

 HepG2 cells were cultured in six-well culture dishes until they reached about 80% confluence. The cells were then treated with Sorafenib (10 μmol/L), Cisplatinum (10 mg/L), or a combination of Sorafenib (10 μmol/L) and Cisplatinum (10 mg/L) for 24 h. Untreated cells acted as the blank control. All the other procedures were performed as previously described (Zhang et al., 2016). The details of the gene primers are summarized in Table 1.

*Western-blot*

HepG2 cells were cultured in six-well culture dishes until they reached about 80% confluence. The cells were then treated with Sorafenib (10 μmol/L), Cisplatinum (10 mg/L), or a combination of Sorafenib (10 μmol/L) and Cisplatinum (10 mg/L) for 24 h. Untreated cells were used as the blank control. All the other procedures were performed as previously described (Gedaly et al., 2013).

*Statistical analysis*

 All analyses were performed using SPSS v.17.0. Data are represented by the mean ± S. ANOVA was used for any comparisons between many groups and the SNK-q test was used for comparison of two groups. Statistical significance was set at P ﹤0.05.

**Figure 1:**
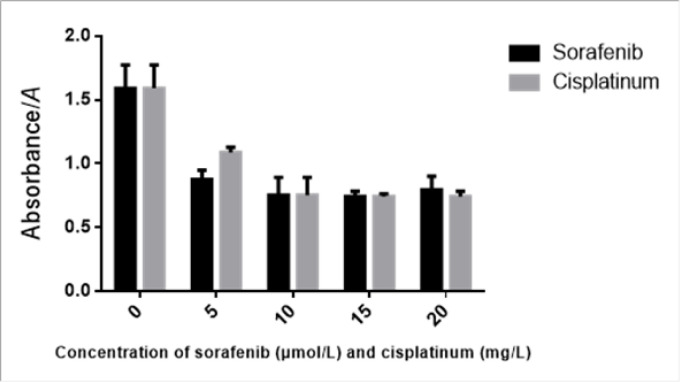
Sorafenib or Cisplatinumum Monotherapy on Inhibition of HepG2 As for sorafenib, *F* =58.48; *P* <0.05; each concentration groups vs control group, *P* <0.05, comparison among 10, 15, 20μmol/L sorafenib groups, *P*>0.05, comparison between 5μmol/L group and other groups, *P*<0.05; As for cisplatinumum, *F* =56.30, *P* <0.05; each concentration groups vs control group, *P* <0.05, comparison among 10, 15, 20mgl/L sorafenib groups, *P*>0.05, comparison between 5mg/L group and other groups, *P*<0.05

**Table 1:** The Types and Sequence of Gene Primers

Genes	Gene sequence	Gene length (bp)
*Β-actin *	F: 5’-GATGAGATTGGCATGGCTTT-3’	268
	R: 5’- CACCTTCACCGTTCCAGTTT-3’	
*AKT*	F: 5’- ACTGCGCTGGACGATAGCTT-3’	107
	R: 5’-AGGACAGCGTGGCTTCTCTC-3’	
*LC3*	F: 5’- CGAGCGCTACAAGGGTGAGA-3’	195
	R: 5’- TCGTAGATGTCCGCGATGGG-3’	
*mTOR*	F: 5’- ATTCAGATCGCTGGCAGCCT-3’	178
	R: 5’- CCCTGTGTTCAGCACCTCCA-3’	

**Figure 2:**
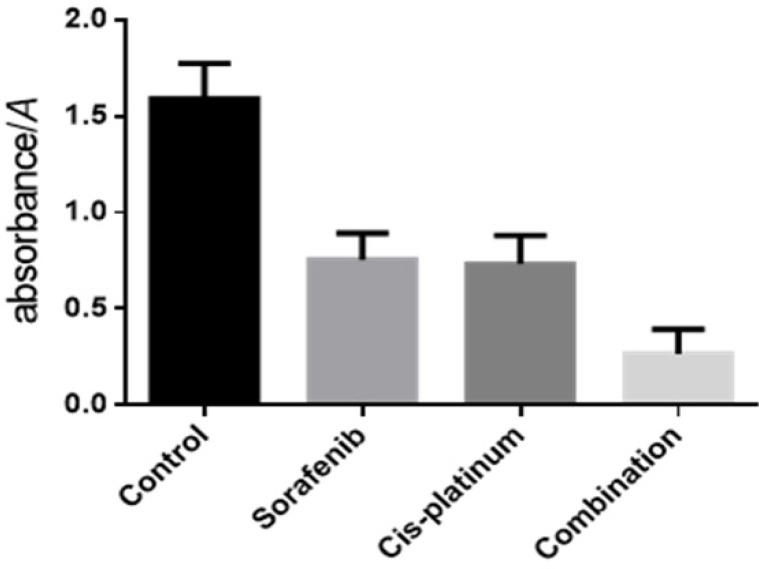
Sorafenib and Cisplatinumum Synegistically Inhibited Proliferation *F*=79.88, *P*<0.05; each groups vs control group, *q* =10.53~16.67, *P* <0.05; mono-drug vs drug combination, *q* =5.87, 6.14, *P* <0.05

**Figure 3:**
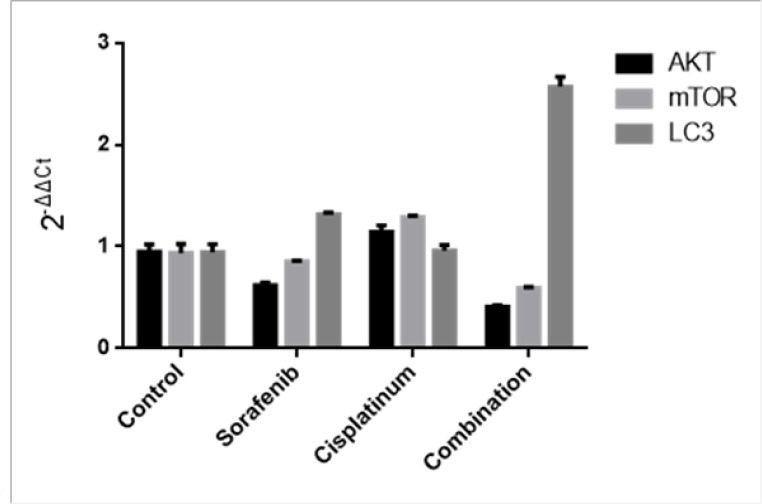
Effects of Different Agents on mRNA of AKT, mTOR, LC3 For the Expression of AKT. *F* =181.32; *P*<0.05; sorafenib vs control group and drug combination vs control group, *q* =10.16~15.77, *P* <0.05; mono-drug vs drug combination,*q* =5.61~19.65; P <0.05; cisplatinum vs control group,* P* >0.05. For the expression of mTOR: *F* =282.10; *P*<0.05; sorafenib vs control group and drug combination vs control group,q =6.50~31.00, *P* <0.05; mono-drug vs drug combination, *q *=74.50~42.00; *P* <0.05; cisplatinum vs control group,P >0.05. For the expression of LC3: *F* =275.70; *P*<0.05; sorafenib vs control group and drug combination vs control group, *q* =14.09~71.36, *P* <0.05; mono-drug vs drug combination,q =57.27~73.18; *P* <0.05; cisplatinum vs control group,P >0.05

**Figure 4:**
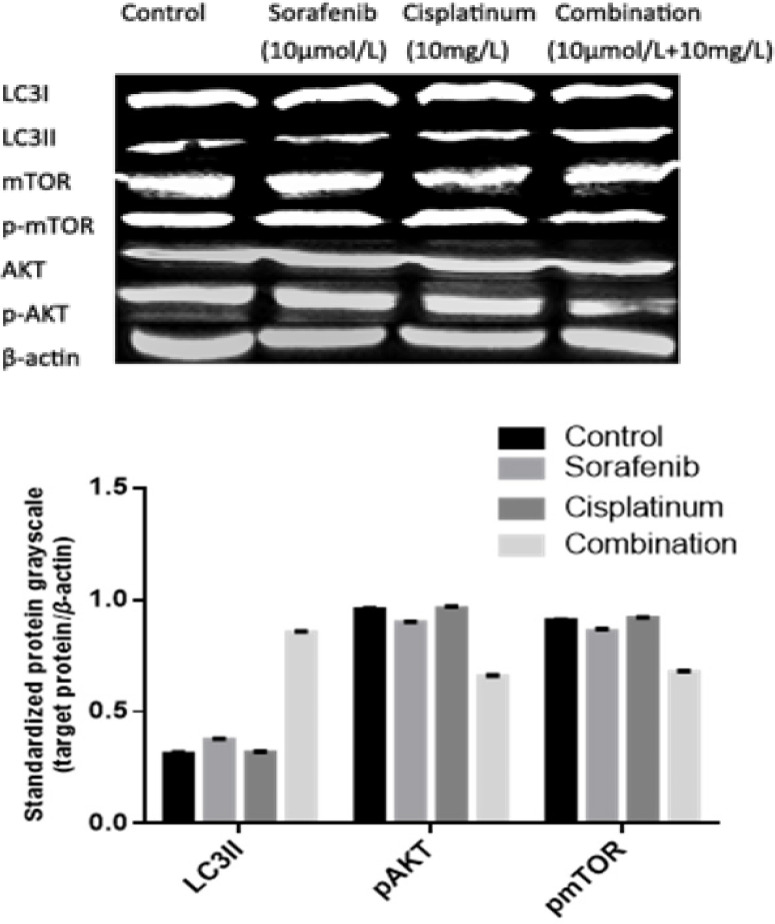
Effects of Different Agents on p AKT, pmTOR, LC3II For the expression of LC3II: F =4104.00; *P*<0.05; drug combination vs control group,q =132.70, *P* <0.05; mono-drug vs drug combination, *q* =117.30~131.30; *P* <0.05, sorafenib vs control group,*q*=15.4, *P* <0.05.but cisplatinum vs control group,q =1.47,P >0.05.For the expression of pAKT : *F* =1236.00; *P*<0.05; drug combination vs control group,*q* =73.70, *P* <0.05; mono-drug vs drug combination,q =58.70~74.70; *P* <0.05; sorafenib vs control group, *q*=14.60, *P* <0.05.but cisplatinum vs control group, *q* =1.00, *P* >0.05. For the expression of pmTOR: *F* =748.00; *P*<0.05, drug combination vs control group, *q* =56.20, *P* <0.05; mono-drug vs drug combination, *q* =44.90~58.60; *P* <0.05; sorafenib vs control group, *q*=11.20, *P* <0.05.but cisplatinum vs control group, *q* =2.40, *P* >0.05

**Table 2:** Absorbance and Cell Survival Rate of Each Group

Groups	Absorbance/A( ±S)	cell survival rate (%)
Control	1.59±0.18	
Sorafenib	0.75±0.14	47.42
Cis-platinum	0.73±0.15	46.04
Combination	0.27±0.13	16.71

*F*=79.88, *P*<0.05; each groups vs control group,*q* =10.53~16.67, *P* <0.05; mono-drug vs drug combination, *q* =5.87, 6.14; *P* <0.0

## Results

*Sorafenib and cisplatinum individually or in combination inhibited HepG2 proliferation*

When Sorafenib and cisplatinum were applied individually they were shown to inhibit HepG2 proliferation in a dose dependent fashion (Figure 1). The cell survival rate of the combination group (Sorafenib 10 μmol/L and cisplatinum 10 mg/L) was significantly more inhibitory than either the Sorafenib (10 μmol/L) or cisplatinum (10 mg/L) groups (Table 2). A combination of Sorafenib (10 μmol/L) and cisplatinum (10 mg/L) resulted in synergistic inhibition of HepG2 cell growth and this effect was shown to be a significant improvement on either treatment individually ( P <0.05) (Figure 2).

*A combination of Sorafenib and cisplatinum inhibited the transcription of key enzymes in the PI3K/AKT/mTOR signaling and autophagy regulation pathways*

AKT, mTOR and LC3 transcription was shown to be repressed following treatment with Sorafenib but not cisplatinum. These effects were shown to be significantly more pronounced when the cells were treated with a combination of Sorafenib and cisplatinum (P<0.05), suggesting that there is a synergistic effect on transcriptional regulation when a combination of these drugs are applied (Figure 3).

*Combination of Sorafenib and cisplatinum inhibits key enzymes in the PI3K/AKT/mTOR and autophagy pathways*

The western blot analysis shows that Akt and mTOR phosphorylation was slightly inhibited, 6.3% and 5.1%, respectively, following Sorafenib (10 μmol/L) treatment but neither protein was effected by cisplatinum (10 mg/L) treatment. Notably Akt and mTOR phosphorylation were significantly inhibited, 31.3% and 25.4%, respectively, by a combination of Sorafenib (10 μmol/L) and cisplatinum (10 mg/L) (P<0.05). The expression levels of LC3II was increased by 175% following treatment with this drug combination which was significantly different from the results of each individual compound (20% by Sorafenib or 1.9% cisplatinum) (P<0.05) (Figure 4).

## Discussion

The incidence rate of hepatocellular carcinoma-HCC-in China continues to increase every year and because of the benign early symptoms most patients are only diagnosed in the terminal stages of the disease. Many of the clinical interventions for HCC have poor therapeutic outcomes, with many also having severe side effects. For instance, the resection rate for terminal-stage HCC is very low and there are severe postoperative complications. Several chemical agents, including cisplatinum, adriacin doxorubicin, fluorouracil and capecitabine are used in clinic interventions but they have played only a minor role in the treatment of HCC. As for radiotherapy, it is predominantly used in the palliative care of HCC patients, and exhibits limited curative potential (Chen and Zhu, 2016). Therefore, the discovery of novel, more effective clinical interventions is imperative for improving HCC therapeutic outcomes.

Sorafenib can be used to extend the median survival duration and time to progression for late or advanced stage HCC, and, at present, Sorafenib is the only effective targeted drug for HCC in clinical use. Sorafenib is a multi-kinase inhibitor known to effect the function of more than a dozen kinases, including serine/threonine kinase Raf, the receptor tyrosine kinase VEGFR and platelet-derived growth factor receptor (PDGFR) (Chaparro et al., 2008). PI3K/AKT/mTOR signaling is one of the most important cellular signal transduction pathways and plays an important role in inhibiting apoptosis and promoting proliferation. Previous investigations (He et al., 2015; Roberto et al., 2012) have found that Sorafenib decreases cellular proliferation via the inhibition of the PI3K/AKT/mTOR signaling pathway. In our study, human hepatocellular carcinoma cells, HepG2, were cultured and treated with varying concentrations of Sorafenib (5 μmol/L, 10 μmol/L, 15 μmol/L and 20 μmol/L) for 24 h. CCK8 assay showed that Sorafenib inhibited HepG2 proliferation in a dose dependent fashion, with a maximum efficiency at 10 μmol/L. We found that both transcription and phosphorylation of AKT and mTOR were inhibited by Sorafenib treatment. All our findings are consistent with the results of the present study. 

At present, most scholars (Kirkin et al., 2009; Alexandre et al., 2012) believe that cell autophagy is a kind of conservative mechanism used to maintain homeostasis in eukaryotic cells, and the process of cell autophagy includes five steps: initiation, nucleation, elongation, maturation, degradation. A decrease in mTOR activity leads to the activation of cell autophagy while LC3 lipidation by PE and membrane insertion is promoted during the third step. LC3–PE then interacts with misfolded and polyubiquitinated proteins via the autophagy receptor proteins (p62, NBR1, or NIX), where after the LC3–PE and autophagy receptor proteins transport these misfolded and polyubiquitinated proteins to the lysosome for degradation (Kirkin et al., 2009; Puissant et al., 2012). Previous reports (Cui et al, 2013) have found that cell autophagy plays an important role in tumor induction and occurrence and that autophagy may be a key strategy for developing new hepatocellular carcinoma treatments. In our study, HepG2 cells were treated with Sorafenib for 24 h which increased both the transcription and translation of the LC3 gene. Taken together these results suggest that Sorafenib induces cell autophagy and decreases cell proliferation by inhibiting PI3K/AKT/mTOR signaling. 

Cisplatinum has been applied as the first-line treatment to many solid tumors. Cisplatinum inhibits cell proliferation by combining with the DNA of tumor cells (Zou et al., 2012). High-dose cisplatinum treatment is known to have many side effects during the actual therapy, including obvious toxic effects on the kidney tissue and bone marrow suppression. At present, there are only a small number of reports describing cisplatinum treatment in hepatocellular carcinoma, and it is not known whether cisplatinum could enhance the effect of Sorafenib on cell autophagy. In our study, HepG2 cells were cultured and treated with various doses of cisplatinum and then evaluated using qPCR, western blot and CCK8 assay. The CCK8 assay showed that cisplatinum inhibits HepG2 proliferation in a dose dependent fashion and we found that cisplatinum treatment had no significant effects on the transcription and phosphorylation of AKT, mTOR, or LC3. However, HepG2 cells treated with a combination of Sorafenib (10 μmol/L) and cisplatinum (10 mg/L) experienced increased expression of autophagy linked genes and reduced cell proliferation suggesting that a combination of these compounds resulted in a synergistic effect improving therapeutic outcomes. 

In conclusion, a combination of Sorafenib and cisplatinum resulted in increased inhibition of HepG2 proliferation, and cisplatinum enhanced the effects of Sorafenib on cell autophagy via the inhibition of the PI3K/AKT/mTOR signaling pathway. However,the specific mechanism underlying this synergistic effect remains unknown and future studies should focus on whether combinations of Sorafenib and cisplatinum exert similar synergistic effects on HCC in vivo.
